# Plasma metabolomic profile varies with glucocorticoid dose in patients with congenital adrenal hyperplasia

**DOI:** 10.1038/s41598-017-17220-5

**Published:** 2017-12-06

**Authors:** Mohammad A. Alwashih, David G. Watson, Ruth Andrew, Roland H. Stimson, Manal Alossaimi, Gavin Blackburn, Brian R. Walker

**Affiliations:** 1 Strathclyde Institute of Pharmacy and Biomedical Sciences, University of Strathclyde, Glasgow, G4 0RE UK; 2 BHF Centre for Cardiovascular Science, Queen’s Medical Research Institute, University of Edinburgh, Edinburgh, EH16 4TJ UK; 3 General Directorate of Medical Services, Ministry of Interior, Riyadh, 13321 Saudi Arabia; 4 Ministry of Health, Riyadh, Saudi Arabia; 5 Glasgow Polyomics, Wolfson Wohl Cancer Research Centre, College of Medical, Veterinary & Life Sciences, University of Glasgow, Garscube Estate Switchback Road, Bearsden, G61 1QH UK

## Abstract

Glucocorticoid replacement therapy is the mainstay of treatment for congenital adrenal hyperplasia (CAH) but has a narrow therapeutic index and dose optimisation is challenging. Metabolomic profiling was carried out on plasma samples from 117 adults with 21-hydroxylase deficiency receiving their usual glucocorticoid replacement therapy who were part of the CaHASE study. Samples were profiled by using hydrophilic interaction chromatography with high resolution mass spectrometry. The patients were also profiled using nine routine clinical measures. The data were modelled by using both multivariate and univariate statistics by using the clinical metadata to inform the choice of patient groupings. Comparison of 382 metabolites amongst groups receiving different glucocorticoid doses revealed a clear distinction between patients receiving ≤5 mg (n = 64) and >5 mg (n = 53) daily prednisolone-equivalent doses. The 24 metabolites which were statistically significantly different between groups included free fatty acids, bile acids, and amino acid metabolites. Using 7 metabolites improved the receiver operating characteristic with area under the curve for predicting glucocorticoid dose of >0.9 with FDR adjusted P values in the range 3.3 E-04 -1.9 E-10. A combination of seven plasma metabolite biomarkers readily discriminates supraphysiological glucocorticoid replacement doses in patients with CAH.

## Introduction

Glucocorticoid replacement therapy is the mainstay of treatment for congenital adrenal hyperplasia (CAH)^1^ and both primary and secondary adrenal insufficiency^2^. Glucocorticoids are also employed commonly in a variety of inflammatory diseases such as rheumatoid arthritis, obstructive lung diseases, and asthma^3^. Although highly efficacious, treatment with glucocorticoids is generally associated with adverse effects such as obesity, hyperglycaemia, hypertension, cardiovascular disease^4^ and osteoporosis^5^_,_ and in children, retarded linear growth. These dose-related adverse effects are observed even amongst CAH patients when the goal is physiological replacement rather than pharmacological anti-inflammatory therapy^6–8^_._ The efficacy of glucocorticoid therapy can be assessed with disease-related endpoints, including adrenal androgen levels in CAH. However, given the narrow therapeutic index, objective monitoring of glucocorticoid toxicity would also be valuable to assist with dose optimisation; unfortunately, the pharmacokinetics of oral glucocorticoids preclude maintenance of blood steroid concentrations within physiological reference ranges, and no sensitive pharmacodynamic biomarkers exist with which to assess glucocorticoid toxicity.

Metabolomic screening has previously been applied to glucocorticoid therapy only for inflammation using urine biomarkers^9^. The aim of this study was to employ metabolomics in plasma samples which were available from patients with CAH^1,6,10^ firstly to establish whether the metabolomics profile varies across the range of glucocorticoid replacement regimes employed in these patients, and secondly to identify metabolites which might be useful for monitoring glucocorticoid toxicity.

## Results

**Figure 1:**
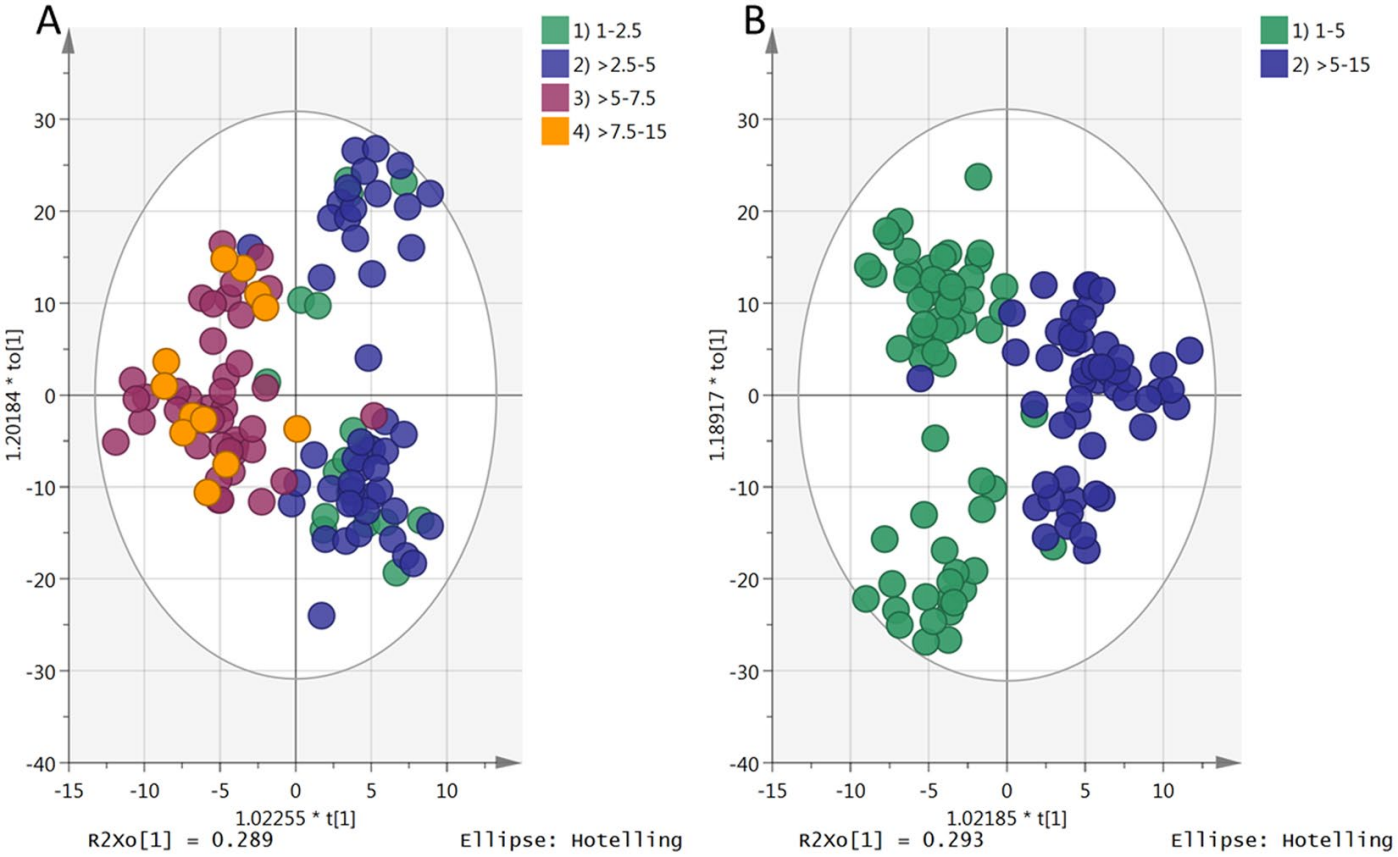
OPLS-DA score plots showing 117 patients with CAH grouped based on their daily doses of glucocorticoid. (**A**) Patients divided into 4 groups by daily prednisolone equivalent dose: 1) patients having 1–2.5 mg (green), 2) >2.5–5 mg (blue), 3) >5–7.5 mg (plum) and 4) >7.5–15 mg (orange). (**B**) Patients divided into 2 groups: 1) 1–5 mg (green-64 samples) and 2) >5–15 mg (blue-53 samples). The later model consists of one predictive x-score component; component t [1] and three orthogonal x-score components to [1–3]. t [1] explains 4.8% of the predictive variation in x, to[1] explains 45.7% of the orthogonal variation in x, R^2^X (cum) = 0.506, R^2^Y (cum) = 1, R^2^ (cum) = 0.829, Goodness of prediction Q^2^ (cum) = 0.657.

In order to examine relationships between glucocorticoid dose and metabolomic profiles, patients were grouped by their daily dose; (1) 1–2.5 mg, (2) >2.5–5 mg, (3) >5–7.5 mg and (4) >7.5–15 mg prednisolone equivalents (Fig. 1). The metabolome profile showed substantial overlap between groups 1&2 and groups 3&4 (Fig. 1A), thus patient doses could not be accurately classified between groups (Table 1). However, a clear difference in metabolomic profile was found between patients receiving 1–5 mg prednisolone equivalents daily (low GC, 64 patients) compared to patients receiving >5–15 mg (high GC, 53 patients) (Fig. 1B). The median (IQR) daily glucocorticoid dose was 3.75 (2.5–5) mg and 7.5 (6.25–7.5) mg for low GC and high GC groups, respectively. There were no statistically significant differences in any of the anthropometric and biochemical measurements between groups (Table 2).

**Table 1:** Data corresponding to Fig. 1 regarding group assignment plus AUROCC for classification.

Group	Samples (n)	Distribution of samples				% of correctly classified samples	AUROCC	*P* CV-ANOVA
**Comparison A**		1–2.5	>2.5–5	>5–7.5	>7.5			
1–2.5	18	0*	17	1	0	0.00%	0.75	2.5E-06
>2.5–5	46	0	43*	3	0	93.48%	0.89	
>5–7.5	41	0	1	40*	0	97.56%	0.9	
>7.5	12	0	1	11	0*	0.00%	0.81	
**Comparison B**		1–5		>5				
1–5	64	64*		0		100%	0.98	1.5E-20
>5	53	2		53*		96%	0.98	

*Number of samples that correctly assigned to the correct group, AUROCC = area under the ROC curve.

**Table 2:** Comparison of anthropometric and clinical measurements between the low (L) (n = 64) and high (H) (n = 53) dose glucocorticoid exposed groups. All measurements were similar between the two groups except for glucocorticoid dose.

Parameter	Glucocorticoid dose group	Mean ± SD	Q1	Median	Q3	FDR-adjusted p value
**Age (y)**	L	36.5 ± 10.8	30.15	35	42.15	0.64
	H	35.4 ± 11.7	25.6	34.85	42.25	
**Weight (kg)**	L	75.7 ± 13.9	65.9	73	83.6	0.76
	H	77.9 ± 17.4	64.4	74.6	89.63	
**Height (m)**	L	1.56 ± 0.08	1.51	1.57	1.62	0.39
	H	1.58 ± 0.08	1.52	1.58	1.64	
**BMI (m/kg** ^2^ **)**	L	30.9 ± 6.04	26.9	30	33.95	0.83
	H	30.84 ± 6.5	26.05	29.35	34.9	
**Systolic blood pressure (mmHg)**	L	118.7 ± 12.1	110.16	117.33	125.8	0.27
	H	122.7 ± 12.6	112.6	123	131.6	
**Diastolic blood pressure (mmHg)**	L	73.53 ± 9.01	68	73.3	79.3	0.21
	H	76.9 ± 8.8	72.6	77	81.08	
^**U**^ **PredEqBNF**	L	3.68 ± 1.3	2.5	3.75	5	1.4E-19
	H	7.52 ± 1.7	6.25	7.5	7.5	
^**U**^ **Serum androstenedione**	L	8.56 ± 19.1	1.475	3.35	5.85	0.42
	H	11.18 ± 15.7	1.7	3.1	15	
^**U**^ **Serum 17-OH progesterone**	L	65.3 ± 98.8	3	11.5	92.45	0.81
	H	82.42 ± 162	4.25	12.55	80.85	
**Female/Male**	L	45/19				
	H	35/18				

^U^p-value based on Mann-Whitney U test (non-parametric), L = 1–5 mg daily prednisolone equivalent, H = >5–15 mg daily prednisolone equivalent, PredEqBNF = daily prednisolone equivalents of glucocorticoids therapies based on British National Formulary.

The OPLS-DA model (Fig. 1B) based on 382 metabolites in 117 patients showed a clear separation between low GC and high GC groups with *P* CV-ANOVA = 7.4E-22. The metabolites which were most different between the two groups are shown in Table 3; the metabolites were refined based area under receiver operating characteristic curve (AUROCC) >0.6^11^. All the metabolites were significantly different between the two groups as judged by the confidence intervals obtained from the jack-knife uncertainty test available in Simca P.

**Table 3:** Putative biomarkers significantly different between the low (L) and high (H) glucocorticoid dose groups.

Metabolites	AUROCC	L:H	VIPpred	VIPortho
Tridecanoic acid(C13:0)	0.63	1:0.9	0.58	0.54
Pentadecanoic acid(C15:0)	0.64	1:1.3	0.98	0.78
Palmitic acid*(C16:0)	0.66	1:1.4	1.06	1.07
Eicosanoic acid (C20:0)	0.65	1:1.4	1.03	0.92
Palmitoleic acid(16:1)	0.77	1:0.7	1.55	0.4
Hydroxyeicosatrienoic acid(20:3)	0.65	1:1.3	1.06	0.75
Docosahexaenoic acid(22:6)	0.65	1:1.2	0.87	0.62
Prostaglandin B1 (C20:2)	0.64	1:1.3	1.02	0.8
Inosine*	0.63	1:0.9	0.69	0.56
Uridine*	0.75	1:0.7	1.21	0.64
Hypoxanthine*	0.73	1:2.4	2.11	1.02
Methionine*	0.73	1:1.2	0.87	0.4
5-L-Glutamyl-taurine	0.65	1:1.6	1.14	0.85
Tryptophan*	0.67	1:1.7	1.59	0.97
Dehydroquinate	0.67	1:1.3	1.02	0.82
3(4-Hydroxyphenyl)pyruvate*	0.75	1:0.5	1.8	0.6
Alpha-N-Phenylacetyl-L-glutamine	0.61	1:0.9	0.55	0.43
4-Hydroxy-2-oxopentanoate	0.65	1:3.5	2.34	1.6
Asparagine*	0.72	1:2.6	2.29	1.14
Threonine*	0.62	1:0.7	1.14	0.56
Keto-glutaramic acid	0.64	1:0.8	0.8	0.65
N-Methylnicotinamide	0.69	1:2.2	1.85	0.85
Octanoylcarnitine	0.66	1:1.4	1.16	0.83
Chenodeoxyglycocholic acid	0.82	1:4.8	6.73	1.46

*Retention time matches standard, AUROCC = area under the ROC curve, VIPpred = predictive value of variable importance in the projection, VIPortho = orthogonal value of variable importance in the projection. VIP values represent the contribution of the metabolite in the variability between the two groups compared to the other metabolites.

**Figure 2:**
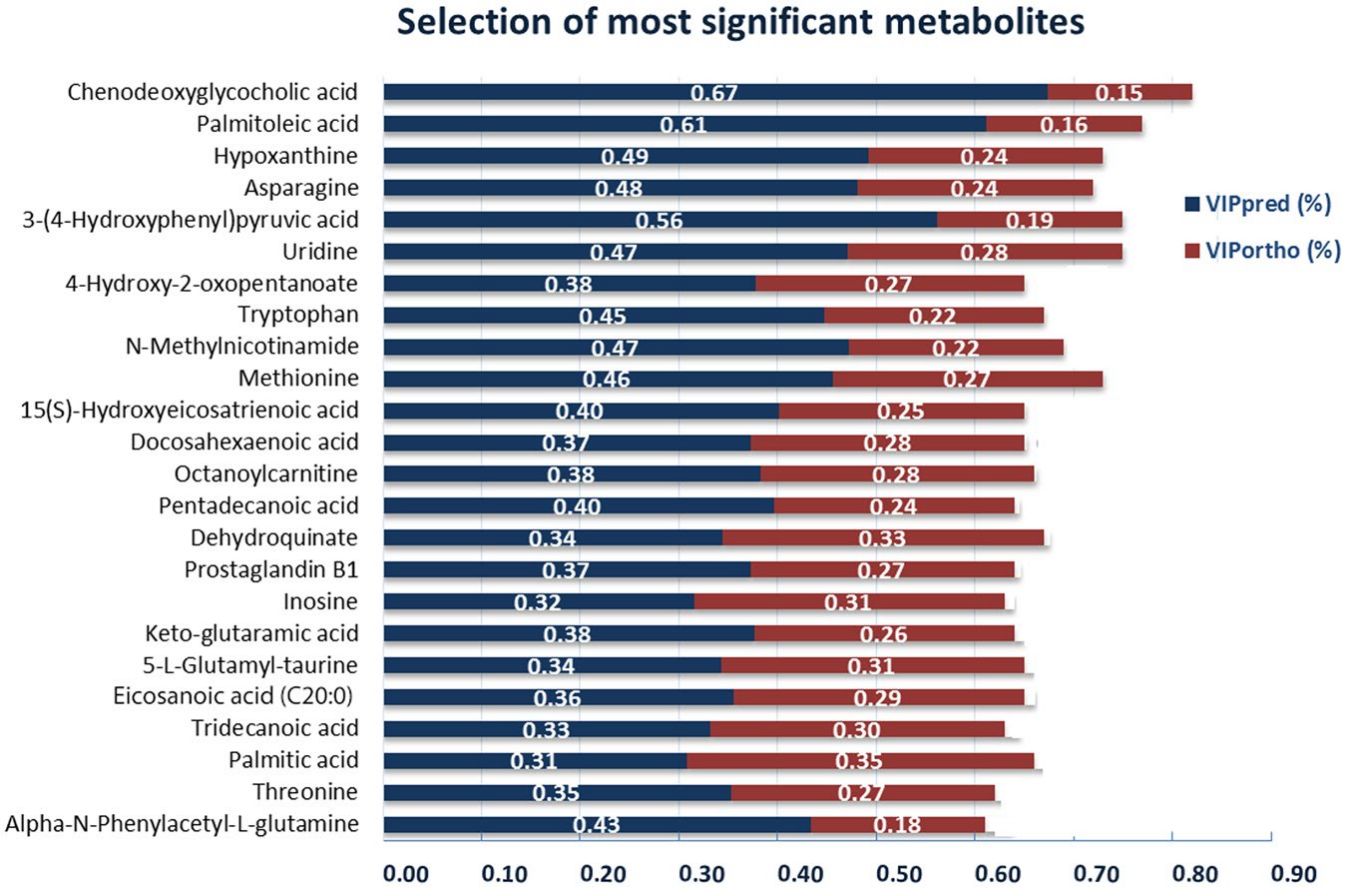
Bars plot shows 24 metabolites (Table 3). Each bar represents a metabolite on y-axis its AUROCC value on the x-axis. Each metabolite bar comprises of two segments; VIPpred (predictive value of variable importance in the projection) (blue) and VIPortho (orthogonal value of variable importance in the projection) (red), their values presented as percentages. A metabolite was included in the final model if it had VIPpred ≥2*VIPortho. Only seven metabolites passed the filter.

**Figure 3:**
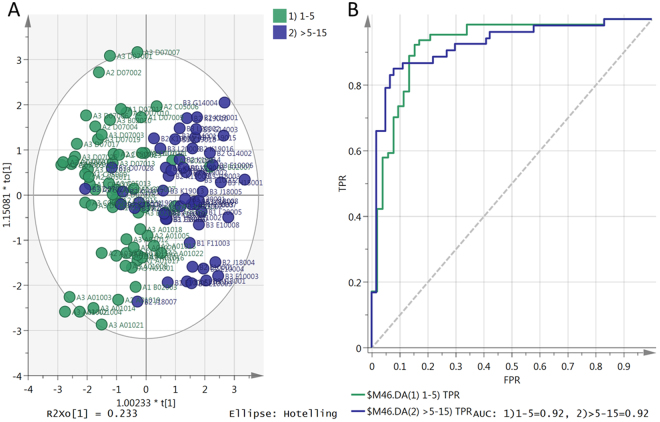
**(A)** OPLS-DA score plot was comprised 7 putative biomarkers (Table 4) quantified in 117 patients. Green observations (64 samples) represent patients receiving a GC dose of 1–5 prednisolone equivalent and the blue observations (53 samples) represent patients receiving GC dose >5–15 mg prednisolone equivalent. The model consists of one predictive x-score components; component t[1] and one orthogonal x-score component to[1]. t[1] explains 33.7% of the predictive variation in x, to[1] explains 23% of the orthogonal variation in x, R^2^X (cum) = 0.57, R^2^Y (cum) = 1, R^2^ (cum) = 0.535, Goodness of prediction Q^2^ (cum) = 0.497. Plot **(B)** showing area under the ROC curve (AUC) of the two groups, x-axis showing (FPR) false positive rate (1-specificity), y-axis showing true positive rate (sensitivity). AUC for 1) 1–5 = 0.92 and 2) >5–15 = 0.92.

The metabolites in Table 3 (24 metabolites) were then refined further by discarding metabolites which did not make a strong individual contribution to predicting glucocorticoid dose, based on their VIPpred versus VIPortho (Fig. 2), resulting in a model (Fig. 3A) based on only seven metabolites (Table 4). These 7 variables in combination produced a combined AUROCC of 0.92 (Fig. 3B). The new model (Fig. 3A) explained more of the variation between low GC and high GC groups (33%) compared to the earlier model (Fig. 1B) which explained only 4.3% of the variation. The majority of the 7 metabolites were positively correlated to glucocorticoid dose; of those, chenodeoxyglycocholate had the highest correlation value (r = 0.76) while N-methylnicotinamide had the lowest correlation value (r = 0.46).

**Table 4:** List of significant biomarkers used to build the OPLS-DA model in Fig. 2.

Putative biomarker	FDR-adjusted p value	L:H	VIP pred	VIP orth	r	99% CI
Asparagine*	4.5E-05	1:2.6	2.29	1.14	0.52	(0.08, 0.34)
Tryptophan*	NA	1:1.6	1.59	0.79	0.53	(0.12, 0.29)
4-Hydroxyphenyl pyruvate*	6.6E-05	1:0.5	1.8	0.6	−0.57	(−0.37, −0.08)
Palmitoleic acid	NA	1:0.7	1.55	0.4	−0.66	(−0.42, −0.1)
Chenodeoxyglycocholate	NA	1:4.8	6.73	1.46	0.76	(0.16, 0.44)
N-Methylnicotinamide	NA	1:2.2	1.85	0.85	0.46	(0.02, 0.34)
Hypoxanthine*	1.8E-05	1:2.4	2.11	1.02	0.51	(0.05, 0.34)

*Retention time matches standard. r = correlation coefficient of a metabolite to high dose of GC.

NA The metabolite is not normally distributed.

## Discussion

Using metabolomic profiling the differences between patients receiving ≤5 and >5–15 mg daily prednisolone equivalent doses of glucocorticoid replacement were shown. This corresponds with the daily dose of prednisolone which is widely regarded as ‘physiological replacement’, at 5 mg daily, suggesting that metabolic profiling is sensitive to supraphysiological glucocorticoid effects. By selecting individual metabolites which in combination could most reliably predict glucocorticoid dose, we identified seven biomarkers which in combination provide an AUROCC of 0.92. These metabolites may form the basis for a ‘kit’ to detect glucocorticoid toxicity. Only three of these biomarkers were normally distributed when a QQ test was applied to the seven biomarkers. However, the OPLSDA model does not rely on normal distribution of markers and the jack-knife uncertainty test for significance^12^ used to confirm confidence intervals is non-parametric.

The glucocorticoid dose-related biomarkers were plausibly associated with glucocorticoid action. Chenodeoxycholic acid is representative of bile acid biosynthesis, which is both regulated by glucocorticoids and may influence glucocorticoid metabolism^13^. Hydroxyphenylpyruvic acid can be converted to tyrosine via transamination, a process which is induced by glucocorticoids^14^. Glucocorticoids induce tryptophan dioxygenase (TDO)^15^ and might be expected to reduce levels of tryptophan and its metabolite N-methylnicotinamide but this is not observed in the current case. TDO has haem at its active centre and enzyme activity is regenerated by coupling with the superoxide anion^16^, since a major source of superoxide is from xanthine oxidase, which converts hypoxanthine via xanthine to uric acid^16^, the elevated hypoxanthine and inosine in the high GC group could indicate inhibition of xanthine oxidase and thus possibly reduced TDO activity. Palmitoleic acid has been used as a plasma marker of stearoyl CoA desaturase (SCD) activity which is required for the secretion of triglycerides by the liver^17^, lower levels, and desaturation of C16:0 to C16:1, in the high GC group are consistent with glucocorticoid inhibition of SCD and induction of fatty liver disease^18^.

In a previous study aromatic amino acids levels were correlated with insulin resistance in 263 lean individuals^19^, tyrosine and phenylalanine were increased in patients receiving high GC dose. The bacterial-derived metabolite 4-Hydroxy-2-oxopentanoate was also higher with insulin resistance^19^. In the current study this metabolite also increases with glucocorticoid dose. In our study C15:0, C16:0, C20:3 and C22:6 fatty acids were all elevated in patients receiving high GC dose while C13:0 and C16:1 fatty acids were reduced (Table 3). Similarly, elevated plasma levels of C16:0, C20:3 and C22:6 were reported in patients with non-alcoholic fatty liver disease (NAFLD)^18^. In our previous study we observed that hydrocortisone increased the levels of a wide range of fatty acids in plasma and insulin opposed this effect^20^. Palmitic acid (C16:0) has a strong positive association with type 2 diabetes, although the odd chain pentadecanoic acid (C15:0) has an inverse association with type 2 diabetes^21^. Urinary excretion of N-methylnicotinamide (NMN), a metabolite of tryptophan which is increased with high GC dose in the current study, has been found to be elevated in type 2 diabetes along with its metabolites the N-methyl pyridine carboxamides, and knock down of nicotinamide N-methyl transferase protects against obesity^22^. Patients with impaired glucose tolerance (IGT) have reduced levels of phenylacetyl-glutamine and increased levels of acylcarnitines and α-ketoglutarate, a pattern indicative of TCA cycle intermediate depletion which interferes with insulin action^23^, as well as reduced tryptophan, xanthine, methionine and nucleotides; patients with diabetes also have a higher plasma level of octanoylcarnitine compared to non-diabetic individuals^24^. We found these diabetes-related metabolites to be altered with glucocorticoid dose (Table 3).

This observational study cannot distinguish metabolites which are directly affected by glucocorticoids from those which are indirectly affected, for example by the documented differences in body composition with variation in glucocorticoid dose, or by differences in efficacy of suppression of adrenal androgens^1,10^. In addition in this large observational study it was not possible to control diet. However, this does not detract from the potential utility of these markers, which are substantially more sensitive than the non-specific clinical indicators presently in use, listed in Table 2. All the 7 candidate biomarkers had AUROC curve values above 0.7 and a high contribution to the separation between the high GC and low GC groups and low within-group variability. Although the current study is limited by use of a single analytical platform, the markers discovered could be used as reliable predictors of supraphysiological GC dose and incorporated into a rapid targeted screen. This is something we will now address in a quantitative manner. There is some commonality between the marker metabolites reported here and those reported in our earlier study^20^. In our previous study increasing the dose of hydrocortisone used increased the levels of docosahexanoic acid, eicosanoic acid (C20:0) and hypoxanthine as observed in the current study. The two studies are not entirely comparable since in the previous study a high and a low dose of corticosteroid was used rather than a gradation of doses as in the current case. What is absent in the current case is a clear effect on branched chain amino acids which in the previous study were elevated by increased HC dose. These metabolites are also established markers of a pre-diabetic state^25^ but are not highlighted as important markers in the current study. The value of a multivariate statistical approach is confirmed in the current study, particularly since the metabolite markers are not normally distributed, and the final OPLSDA model is very strong considering that the seven biomarkers can be used to largely distinguish between the two groups in this large co-hort.

## Materials and Methods

Experimental details for sample preparation and analysis are given in supplementary material along with details for data extraction and metabolite identification.

### Patient recruitment

The UK Congenital adrenal Hyperplasia Adult Study Executive (CaHASE) cohort is a cross-sectional study of adult CAH patients (aged ≥18 years) recruited from 17 specialized endocrine centres across the UK. The study protocol was approved by West Midlands research ethics committee (MREC/03/7/086) and registered with ClinicalTrials.gov (NCT00749593) and has been previously published in detail^10^. All participants gave written informed consent. All methods were performed in accordance with the relevant guidelines determined by the protocols approved by the ethics committee. This study was not a clinical trial but was an observational clinical study and therefore is not categorised as a clinical trial and is not registrable as one.

### Clinical Procedures

Participants attended the research unit of their respective centre after an overnight fast having taken their regular medication, followed by medical history, physical examination (height, weight, blood pressure) and blood sampling (including for 17-hydroxyprogesterone (17OHP), androstenedione). All laboratories participate in the UK NEQAS scheme for quality control of steroid assays. Inclusion criteria for the metabolomics analysis were as follows: known 21-hydroxylase deficiency; additional serum sample collected at time of recruitment; full anthropometric and biochemical data available for each participant. Samples from 117 patients were used for metabolomics analysis; subjects were treated with hydrocortisone, prednisolone and dexamethasone or combination therapy. Glucocorticoid therapies were converted to daily prednisolone equivalents based on the relative potencies of the steroids reported in the British National Formulary (PredEqBNF)^26^.

### Statistical Analysis

The methods used for statistical analysis are described in supplementary material and also in our previous publication^20^.

### Data Availability Statement

The datasets generated during and/or analysed during the current study are available from the corresponding author on reasonable request.

## Electronic supplementary material

Supplementary information
