# Supplementary material for: Plasma metabolomic profile varies with glucocorticoid dose in patients with congenital adrenal hyperplasia
Source: Sci Rep. 2017 Dec 6;7:17092. doi: 10.1038/s41598-017-17220-5 (PMC5719028; doi:10.1038/s41598-017-17220-5)
Supplement: Supplementary file 1 — Supplementary information [file 41598_2017_17220_MOESM1_ESM.pdf]

Plasma metabolomic profile varies with glucocorticoid dose in patients with congenital adrenal hyperplasia

Mohammad A. Alwashih[1,3], David G. Watson*[1], Ruth Andrew[2], Roland H Stimson[2], Manal Alossaimi[1,4], Gavin Blackburn[5] and Brian R. Walker[2]

[1] Strathclyde Institute of Pharmacy and Biomedical Sciences, University of Strathclyde, Glasgow G4 0RE, UK.

[2]BHF Centre for Cardiovascular Science, Queen's Medical Research Institute, University of Edinburgh, Edinburgh EH16 4TJ, UK.

[3] General Directorate of Medical Services, Ministry of Interior, Riyadh 13321, KSA.

[4] Ministry of Health, Riyadh, KSA.

[5] Glasgow Polyomics, Wolfson Wohl Cancer Research Centre, College of Medical, Veterinary & Life Sciences, University of Glasgow, Garscube Estate Switchback Road, Bearsden G61 1QH, UK.

Materials and Methods

Chemicals and materials

HPLC grade acetonitrile (ACN) was from Fisher Scientific, UK. HPLC grade water was produced by a Direct-Q 3 Ultrapure Water System from Millipore, UK. AnalaR grade formic acid (98%) was from BDH-Merck, UK. Ammonium carbonate and ammonium acetate were from Sigma-Aldrich, UK.

Sample Preparation

Samples were stored at -80°C and thawed at ambient temperature before further preparation. A pooled sample was prepared by taking 20 µl of 10 randomly selected samples. Metabolites were extracted by transferring 200 µl of sample to an Eppendorf tube with addition of 800 µl of ACN. After vortexing the samples were centrifuged at 8000 RPM for 10 min. The supernatant was then collected into a HPLC vial for LC-MS analysis.

LC-MS Analysis

Sample analysis was carried out on an Accela 600 HPLC system combined with an Exactive (Orbitrap) mass spectrometer (Thermo Fisher Scientific, UK). An aliquot of each sample solution (10 µl) was injected onto a ZIC-pHILIC column (150 × 4.6 mm, 5 µm; HiChrom, Reading, UK) with mobile phase A (20 mM ammonium carbonate in HPLC grade water (pH 9.2)), and B (HPLC grade acetonitrile). The gradient was formed by decreasing the percentage of B from 80% to 20% over 30 minutes followed by washing the column at 5% of B for 5 minutes and finally re-equilibrating the column at 80% of B for 10 minutes [1]. Samples were submitted in random order for LC-MS analysis, and pooled quality control samples were injected after every 20 samples to monitor the stability of the instrumentation. Standard mixtures containing authentic standards for 220 compounds were run in order to calibrate the columns. The Exactive Orbitrap (Thermo Fisher Scientific, Hemel Hempstead, UK) was operated in both positive and negative modes set at 50,000 resolution and controlled by Xcalibur version 2.1.0 (Thermo Fisher Corporation, UK). The mass scanning range was m/z 75–1200;

the capillary temperature was 320 °C; and the sheath and auxiliary gas flow rates were 50 and 17 arbitrary units, respectively.

Data Extraction

Raw LC-MS files were converted to mzXML (ProteoWizard) and separated into ESI positive and negative. Converted files were then processed with open source MzMatch (http://mzmatch.sourceforge.net/) and the identification of putative metabolites was made via the macro-enabled Excel file, Ideom (http://mzmatch.sourceforge.net/ideom.html). Thus metabolites were identified to MSI levels 2 or 3 [2] according to either exact mass (< 3 ppm deviation) or exact mass plus retention time matching to a standard.

Data Analysis

Softwares used

All data processing, including data visualisation, biomarker identification, diagnostics and validation was implemented using SIMCA software v.14 (Umetrics AB, Umeå, Sweden) for multivariate analysis[3]. For univariate analysis, Metaboanalyst 3.0 (**www.metaboanalyst.ca**) [4] to get FDR adjusted p-value and AUC for each metabolite. And IBM SPSS Statistics software package version 22.0 (IBM SPSS, Chicago, IL) were employed to test statistical difference between anthropometrics measurements in low compared to high GC dose**.** The normality of the anthropometrics and clinical data was examined statistically by Shapiro-Wilk test; if this was violated then Mann Whitney test was employed.

Data visualisation and biomarkers identification

Orthogonal projections to latent structures-discriminant analysis (OPLS-DA), a supervised model, was employed to examine the differences between groups while neglecting the systemic variation, It links the metabolomic variability among samples either to the intervention (predictive) or to a systematic variation (orthogonal) [5]. Variable importance in the projection (VIP) was employed to assess the contribution of each variable in the observed metabolomics change to a given model compared to the rest of variables [6,7]. The average VIP is equal to 1; thus a variable with VIP larger than 1 has more contribution in explaining *y* and vice versa [6,7]. The 99% confidence interval was calculated for each

metabolite based on jack-knife of uncertainty which estimates the prediction error rate based on the cross validation rule used [8]. Correlation coefficient of a metabolite to high dose of GC was used to evaluate the reliability of a metabolite, reliable metabolites > |5| [9]. Metabolites were then filtered based on their 99% CI so that all metabolites with CIs crossing the zero point were filtered out. The metabolites used to build the final model were selected based on VIPpred ≥ 2*VIPortho resulting an OPLSDA model based on 7 metabolites.

Diagnostics and validation

R^2 and Q^2 were employed as diagnostic tools for supervised and unsupervised models. The R^2 represents the percentage of variation explained by the model while Q^2 indicates cross validated R^2 [5]. Model validity was also assessed using cross validated ANOVA (CV-ANOVA) which corresponds to H_0 hypothesis of equal cross validated predictive residual of the supervised model in comparison with the variation around the mean [10]. The area under receiver operating characteristic curve (AUROCC) was used to assess the accuracy of the classifier with a rough guide as follow; 0.9–1.0 = excellent; 0.8–0.9 = good; 0.7–0.8 = fair; 0.6–0.7 = poor; 0.5–0.6 = fail [11].

Q-Q plot

Quantile–Quantile (Q-Q) plot is one of the graphical methods used to evaluate the data distribution [12]. The theoretical quantiles (X-axis) were plotted against the samples quantiles (Y-axis) using Minitab 18. Thus, the Q-Q plot was able to evaluate the normality of the distribution of the measurements in comparison with the theoretical probability distribution. In case where the two quantiles were similar, the distributions were also similar and the points fell close to the straight line (x=y). If the two quantiles were different, this indicated that there was a difference in the distribution and the points appeared far from x=y line [13] and the data was not normally distributed.

References

1 Zhang, T. *et al.* Application of Holistic Liquid Chromatography-High Resolution Mass Spectrometry Based Urinary Metabolomics for Prostate Cancer Detection and Biomarker Discovery. *PLoS One* **8**, e65880, doi:10.1371/journal.pone.0065880 (2013).

2 Sumner, L. W. *et al.* Proposed minimum reporting standards for chemical analysis Chemical Analysis Working Group (CAWG) Metabolomics Standards Initiative (MSI). *Metabolomics* **3**, 211-221, doi:10.1007/s11306-007-0082-2 (2007).

3 Zhang, R. *et al.* Metabolomic Profiling of Post-Mortem Brain Reveals Changes in Amino Acid and Glucose Metabolism in Mental Illness Compared with Controls. *Comput Struct Biotechnol J* **14**, 106-116, doi:10.1016/j.csbj.2016.02.003 (2016).

4 Xia, J., Sinelnikov, I. V., Han, B. & Wishart, D. S. MetaboAnalyst 3.0--making metabolomics more meaningful. *Nucleic acids research* **43**, W251-257, doi:10.1093/nar/gkv380 (2015).

5 Kirwan, G. M. *et al.* Building multivariate systems biology models. *Anal Chem* **84**, 7064-7071, doi:10.1021/ac301269r (2012).

6 Eriksson, L., Byrne, T., Johansson, E., Trygg, J. & Vikstrom, C. in *Multi- and Megavariate Data Analysis: Basic Principles and Application* Ch. 503, 455-456 (MKS Umetrics AB, 2013).

7 Chong, I.-G. & Jun, C.-H. Performance of some variable selection methods when multicollinearity is present. *Chemometrics and Intelligent Laboratory Systems* **78**, 103-112, doi:10.1016/j.chemolab.2004.12.011 (2005).

8 Efron, B. & Gong, G. A Leisurely Look at the Bootstrap, the Jackknife, and Cross-validation. *The American Statistician* **37**, 36-48 (1983).

9 Wiklund, S. *et al.* Visualization of GC/TOF-MS-based metabolomics data for identification of biochemically interesting compounds using OPLS class models. *Analytical Chemistry* **80**, 115-122, doi:10.1021/ac0713510 (2008).

10 Eriksson, L., Trygg, J. & Wold, S. CV-ANOVA for significance testing of PLS and OPLS (R) models. *J Chemometr* **22**, 594-600, doi:10.1002/cem.1187 (2008).

11 Xia, J., Broadhurst, D. I., Wilson, M. & Wishart, D. S. Translational biomarker discovery in clinical metabolomics: an introductory tutorial. *Metabolomics* **9**, 280-299, doi:10.1007/s11306-012-0482-9 (2013).

12. Vinaixa, M. *et al.* A guideline to univariate statistical analysis for LC/MS-based untargeted metabolomics-derived data. *Metabolites* **2**, 775-795 (2012).

13. Lee, J. *et al.* Quantile normalization approach for liquid chromatography–mass spectrometry-based metabolomic data from healthy human volunteers. *Analytical Sciences* **28**, 801-805 (2012).
